# Dogs Harbor *Leishmania braziliensis* and Participate in the Transmission Cycle of Human Tegumentary Leishmaniasis

**DOI:** 10.3390/pathogens12080981

**Published:** 2023-07-27

**Authors:** Jamile Lago, Deborah Fraga, Lívia Coelho, Matheus Silva de Jesus, Bruna Leite, Guilherme L. Werneck, Sérgio Arruda, Ednaldo Lago, Edgar M. Carvalho, Olivia Bacellar

**Affiliations:** 1 Immunology Service, Professor Edgard Santos University Hospital Complex, Federal University of Bahia, Salvador 40110-160, BA, Brazil; jamilelago27@gmail.com (J.L.); ednaldo.lago@ig.com.br (E.L.); imuno@ufba.br (E.M.C.); 2 Post-Graduate Course in Health Sciences, Federal University of Bahia Medical School, Salvador 40026-010, BA, Brazil; 3 Gonçalo Moniz Institute (IGM), Fiocruz, Salvador 40296-710, BA, Brazil; deborah.fraga@fiocruz.br (D.F.); livia.vetcoelho@gmail.com (L.C.); silvamath@outlook.com (M.S.d.J.); brunamml@yahoo.com.br (B.L.); sergio.arruda@fiocruz.br (S.A.); 4 Department of Epidemiology, State University of Rio de Janeiro, Rio de Janeiro 20950-000, RJ, Brazil; gwerneck@gmail.com; 5 Institute for Public Health Studies, Federal University of Rio de Janeiro, Rio de Janeiro 22290-240, RJ, Brazil; 6 Instituto Nacional de Ciência e Tecnologia em Doenças Tropicais (INCT-DT), Ministério da Ciência e Tecnologia e Inovação (MCTI), CNPq, Salvador 40110-160, BA, Brazil

**Keywords:** canine tegumentary leishmaniasis, canine subclinical infection, *Leishmania (Viannia) braziliensis*, human American tegumentary leishmaniasis

## Abstract

Dogs play an important role in transmission of *Leishmania infantum*, but epidemiologic and clinical studies of canine tegumentary leishmaniasis (CTL) are scarce. In an endemic area of human American tegumentary leishmaniasis (ATL) caused by *Leishmania braziliensis,* we determine the prevalence and incidence of both CTL and subclinical (SC) *L. braziliensis* infection in dogs and evaluated if the presence of dogs with CTL or SC *L. braziliensis* infection is associated with the occurrence of human ATL. SC infection in healthy animals and CTL in animals with ulcers were determined by PCR on biopsied healthy skin or on ulcers or by detecting antibodies against soluble leishmania antigen. We compared the occurrence of human ATL in homes with dogs with CTL or SC infection with control homes without dogs or with dogs without CTL or SC infection. The prevalence of SC infection was 35% and of CTL 31%. The incidence of SC infection in dogs was 4.6% and of CTL 9.3%. The frequency of ATL in humans was 50% in homes with infected dogs and 13% in homes without *L. braziliensis* infection in dogs. CTL and SC infection is highly prevalent, and dogs may participate in the transmission chain of *L. braziliensis*.

## 1. Introduction

Leishmaniasis is classified as visceral leishmaniasis (VL) and tegumentary leishmaniasis (TL). Approximately 20 *Leishmania* species may cause human disease. In the New World, VL is caused by *Leishmania infantum*, and TL is caused mainly by *Leishmania Viannia braziliensis*, *Leishmania Viannia guyanensis*, and *Leishmania mexicana mexicana.* American tegumentary leishmaniasis (ATL) affects the skin and mucosa, and it is transmitted by an infected female sand fly vector. In humans, the consequences of the infection range from unapparent or subclinical (SC) infection to severe ulcerated lesions. Human ATL represents an important public health problem due to its incidence, risk of deformities, and consequent presence of stigmatizing lesions. Moreover, it has a social and economic impact since it can be considered an occupational disease in most cases [1,2]. ATL can be present in different clinical forms such as cutaneous (CL), mucosal (ML), disseminated (DL), and diffuse cutaneous leishmaniasis. CL is documented in more than 90% of the ATL cases, and *Leishmania Viannia braziliensis* is the most prevalent species in Latin America. The life cycle of *L. (V.) braziliensis* includes a few different wild reservoirs, such as rats and marsupials and domestic mammals such as dogs, horses, and donkeys, as well as several vector species. Due to the small number of wild animals infected with *L. braziliensis* found in endemic areas, the potential role of domestic animals as reservoirs has been emphasized [3,4]. The role of domestic dogs in the transmission cycle of ATL has been considered important because of the proximity of dogs and humans [5].

While little is known about canine tegumentary leishmaniasis (CTL) and the role of dogs as a reservoir of *L. braziliensis,* there is clear evidence of the role of dogs in the epidemiology of visceral leishmaniasis (VL) caused by *L. infantum*. The prevalence of canine and human VL in the same endemic areas has been documented. Strategies for the prevention and control of VL in Brazil dictated by the Ministry of Health include early diagnosis and adequate treatment of human cases, use of insecticides and sanitary measures in the residential environment to reduce vector density, as well as identification and elimination/treatment of dogs with VL [6,7]. Moreover, several vaccines against VL for dogs are available on the market [8,9,10,11].

The finding of CTL or the SC *L. braziliensis* infection in regions where human ATL cases also occur supports to the idea that dogs play a role in the transmission cycle of ATL [12]. In fact, we have recently shown the presence of both symptomatic and SC *L. braziliensis* infection in dogs in a highly endemic area for human ATL [13]. However, the infection of dogs by *L*. *braziliensis* and CTL occurrence has been poorly investigated. Understanding that dogs are an important reservoir of *L. braziliensis*, as observed in VL caused by *L. infantum*, creates the possibility of evaluating in the future the impact of the control of CTL caused by *L. braziliensis* in the occurrence of ATL. The objective of the present study was to determine the incidence and prevalence of CTL caused by *L. braziliensis* and to evaluate the occurrence of human CL in homes with or without dogs with CTL or SC *L. braziliensis* infection.

## 2. Materials and Methods

### 2.1. Study Area

This study was conducted in the village of Corte de Pedra, which is part of the municipality of Tancredo Neves, and in the village of Nova Esperança, which is part of the municipality of Wenceslau Guimarães, both located in the southeastern region of Bahia, Brazil, where ATL caused by *L. braziliensis* is endemic. These villages are characterized by isolated areas of secondary forest, with agricultural activities providing the main source of income for the population. Agricultural work increases exposure to *L. braziliensis* through contact with *Nyssomyia whitmany* and *Nyssomyia intermedia*, the main vectors of *L. braziliensis* in the region [14,15]. These vectors are also present in and around homes, including the peridomicile, which increases the transmission of *Leishmania* infection [16].

### 2.2. Type of Study

This study has two distinct objectives: first, to determine the prevalence and incidence of subclinical *L. braziliensis* infection in dogs, and, second, to understand the relationship between the occurrence of human CL and the presence of dogs infected with *L. braziliensis* in the same homes. To evaluate the prevalence of SC *L. braziliensis* infection and CTL in the village of Corte de Pedra, we performed a cohort study to determine the number of dogs without the disease or with CTL in September of 2018. The incidence of canine infection caused by *L. braziliensis* and CTL was conducted from September of 2018 to August of 2019. For the second objective, a cohort study was conducted in the village of Nova Esperança to determine the frequency of human ATL in households with and without *L. braziliensis* infected dogs or dogs with CTL in the same houses. In this study, cases were defined as homes with dogs presenting CTL or SC infection, and controls were houses without dogs or with dogs without CTL or SC infection.

### 2.3. Study Design for the First Objective

In order to determine the incidence and prevalence of CTL in the village of Corte de Pedra, seven rural areas of the village within a five-kilometer radius of the health post were chosen to be evaluated in the study. All dogs (N = 214) living in the houses of these seven neighborhoods participated in the study. *L. braziliensis* infection in healthy animals was determined by detection of *L. braziliensis* DNA in biopsied healthy skin obtained from the right paleta by PCR or by detecting antibodies against soluble *L. braziliensis* antigen (SLA) by ELISA. The occurrence of CTL in animals with ulcers indicating possible leishmaniasis infection was determined by the presence of *L. braziliensis* DNA in biopsied tissue on the border of the ulcer or the presence of anti-leishmanial antibodies. In order to determine the incidence of CTL and SC infection, animals without the disease and evidence of *L. braziliensis* infection at the baseline time (September 2018) were followed up for a period of one year. Dogs were then reevaluated to determine the occurrence of CTL by documentation of a typical cutaneous ulcer, plus detection of *L. braziliensis* DNA by PCR or anti-leishmanial antibodies.

### 2.4. Study Design for the Second Objective

To determine the association between CTL and human CL, a case control study was performed in the village of Nova Esperança, municipality of Wenceslaus Guimarães, located 51 km from the village of Corte de Pedra. This village was chosen because an outbreak of human CL was documented in this area in 2015, while in the village of Corte de Pedra most people had already had CL in the past; consequently, new cases of human CL are less frequent. To determine if there was an association between CTL and human ATL, visits were made to all the homes in the village between September of 2018 and August of 2019. A survey was performed to determine the total number of dogs in the homes of Nova Esperança residents and the number of homes with dogs with SC *L. braziliensis* infection or with CTL. Cases were defined as houses (N = 19) with dogs with SC *L. braziliensis* infection or with CTL. Controls were defined as houses (N = 15) within a radius of 200 m of the cases’ houses that did not have dogs or that had dogs but without SC infection or CTL. The number of humans with CL from 2018 to 2022 in cases and control houses were recorded.

### 2.5. Diagnosis of SC Canine L. braziliensis Infection, CTL, and Human ATL

The SC *L. braziliensis* infection in dogs was determined by identification of the *L. braziliensis* DNA in tissue biopsy of healthy skin from the right paleta of dogs without evidence of lesions, or by detection of antibodies to *L. braziliensis* using ELISA [17,18]. CTL was diagnosed by the presence of ulcerated lesions and detection of *L. braziliensis* DNA in the lesion, or a positive serologic test in the absence of previous history of CTL. To perform the serologic test, animals were immobilized. After clinical examination, venous blood was collected from the lateral safena vein. A skin biopsy from the ulcer periphery or of healthy skin from the right paleta was performed with 3 mm punch. The material collected from the biopsy was placed in a tube containing RNA for the later detection of *L. braziliensis* DNA, and the serum was put in Eppendorf tubes and stored at −20 °C. Subjects living in control houses with typical CL or a possible CL ulcer were referred to the Corte de Pedra health clinic, where the diagnosis was performed by detection of *L. braziliensis* DNA with PCR in biopsied tissue from the ulcer or by documentation of amastigotes of *L. braziliensis* in histopathologic studies.

### 2.6. PCR for Identification of Leishmania DNA

The detection of *Leishmania* through real-time PCR was performed after obtaining the genomic DNA from the biopsies with TaqMan/probe assays, Custom TaqMan Gene Expression Assay (Applied Biosytems Inc., Foster City, CA, USA), using primers based on KDNA3 sequences, specific for *L. braziliensis*, and a melt curve analysis [19].

### 2.7. Soluble Leishmania Antigen (SLA), Serologic Test and Histopathology

The SLA was obtained from an isolate of L. braziliensis as previously described by [20]. Antibody detection to SLA was performed by an in-house ELISA, with *L*. *braziliensis* SLA, as previously described [21,22,23]. Sera from uninfected dogs living outside an endemic area of *L. braziliensis* transmission were used as control. Absorbances higher than the mean plus 2 standard deviations (SD) of the negative control dogs’ sera were considered positive. Amastigotes detected in biopsied tissue were later stained with hematoxylin and eosin.

### 2.8. Statistical Analysis

The distribution of continuous variables such as age and number of lesions were expressed as mean and standard deviation (X ± SD) and analyzed using Student’s t test. Continuous variables that did not follow a normal distribution were analyzed by a Mann–Whitney test. The proportions as frequency of homes with human CL in both the case and control residences were analyzed by the Fisher exact test. Odds ratio (OR) was calculated to evaluate the association between human CL cases and homes with positive dogs. A *p*-value < 0.05 was considered statistically significant.

## 3. Results

**Figure 1:**
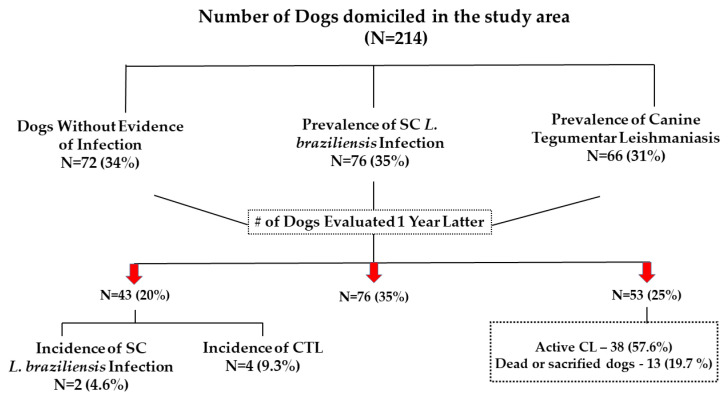
Prevalence of subclinical infection and canine tegumentary leishmaniasis in the village of Corte de Pedra.

The prevalence and incidence of canine SC infection and CTL in 214 dogs from seven different rural areas in the village of Corte de Pedra are shown in Figure 1.

SC *L. braziliensis* infection was detected in 76 (35%) and CTL in 66 (31%) of the animals. A total of 72 (34%) dogs had no evidence of *L. braziliensis* infection at the baseline. After one year, they were reassessed and evaluated for the presence of SC infection or CTL. At the time of the reevaluation, only 43 dogs were found. The incidence of SC *L. braziliensis* infection was 4.6, and 9.3% for CTL. Among the 29 dogs lost after one year at the time of follow-up, their owners said that two dogs disappeared, and the other 27 animals were sacrificed by the neighbors because they had developed typical ulcers indicating CL. Therefore, the incidence of CL was underestimated, and if these 27 sacrificed dogs were added to the two animals that were detected, the incidence of development of CTL would be 40.2%.

The demographic features and positive results of the diagnostic tests in animals with SC infection or CTL detected in seven rural areas of Corte de Pedra are shown in Table 1.

There was no age difference between the two groups of animals, but the frequency of male dogs with CTL was higher (*p* ˂ 0.05) than in the general population of dogs with SC *L. braziliensis* infection. SLA antibodies were detected in all dogs with CTL and with the SC infection, and DNA of *L. braziliensis* was detected in 47 (71%) of the dogs with CTL and in 3.9% of the animals with the SC infection.

Taking advantage of the presence of 66 dogs with CTL and the need to better describe the clinical features and the natural history of this disease, as these animals did not receive therapy, the animals were evaluated in a subsequent follow-up. The prevalence of SC infection after one year was 35%, and all remained with SC infection.

Among the 66 dogs that presented CTL at baseline, 33 (50%) had one ulcer, 20 (30.3%) had two lesions, 6 (9%) had three lesions, and the other 7 presented four or more lesions. Moreover, the ulcers were most frequent on the ears (52%) and scrotum (30%), and in 10% of the dogs on both the ears and scrotum (data not shown). After the 1-year follow-up, the majority (57.6%) of the 66 dogs who had CTL in 2018 remained with the active disease. Additionally, 22.7% self-healed their ulcers, and 19.7% were dead or were sacrificed (Table 1).

Table 2 presents a description of the canine and human population evaluated in the village of Nova Esperança. The prevalence of human CL in Nova Esperança was 36%. For CTL, it was 14.7%. The SC *L. braziliensis* infection in dogs was 22.7%.

**Figure 2:**
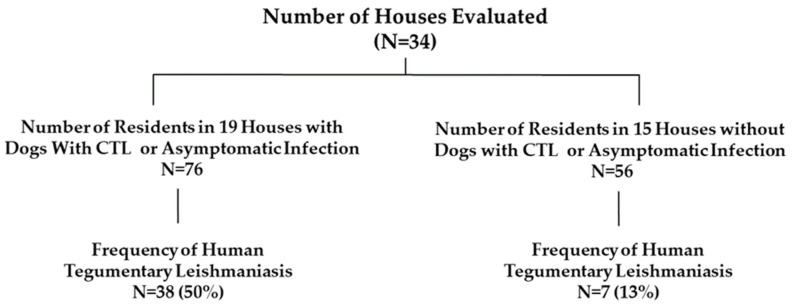
Frequency of human tegumentary leishmaniasis in houses with or without dogs with subclinical infection and with or without dogs with canine tegumentary leishmaniasis.

The frequency of human CL in the homes with or without dogs with *L. braziliensis* infection is shown in Figure 2. In 19 homes that had dogs with CTL or with SC infection, 38 (50%) among the 76 residents of these homes developed CL between September 2018 and August 2022. In contrast, only 7 (13%) people in homes without dogs or with dogs but without CTL or SC *L. braziliensis* infection developed CL in the 4-year period. People living in houses with dogs with CTL or with SC infection presented 4 times more chance of having CL than others in homes without dogs or dogs that did not test positive for CTL (RR = 4.00, IC 95% 1.93–8.29, *p* = 0.0002).

## 4. Discussion

Occurrences of CTL in areas of *L. braziliensis* transmission have been reported, but most of them are series of cases and the species of *Leishmania* was not determined [24]. In a recent survey in the endemic area of Corte de Pedra, we described the clinical manifestations and the diagnosis of CTL in 61 animals who had lesions indicating a possibility of leishmaniasis. In this previous study, we showed that in addition to CTL, there was also evidence of dogs with SC *L. braziliensis* infection based on the detection of DNA of *L. braziliensis* on healthy skin or positive serologic tests for SLA. However, there are a lack of studies that attempt to determine the incidence of CTL and SC *L. braziliensis* infection in dogs. The natural history of CTL in the region is still not defined, nor is the impact of CTL and its role in the occurrence of human CL. In the present study, we documented a prevalence of 31% of CTL and 35% of SC *L. braziliensis* infection, and an incidence of 9.3% and 4.6%, respectively. Moreover, we found an association between the presence of dogs with an infection from the disease caused by *L. braziliensis* and human CL.

While the epidemiology of CTL is poorly studied and data about prevalence and incidence of CTL have not yet been determined, there are many studies about canine VL. It is known that VL and ATL usually do not occur in the same area [25,26,27]. In two northeastern Brazil states, the prevalence and incidence of canine VL caused by *L. infantum* ranged from 3 to 5% and 21 to 25%, respectively [28,29]. in more recent studies in Bahia, Brazil, the prevalence and incidence of canine VL were documented at 56 and 31%, respectively [30]. Here, we showed a high prevalence and incidence of CTL in an area of *L. braziliensis* transmission where a high number of human ATL cases have been reported since 1987 [26].

The demographics and clinical features of CTL caused by *L. braziliensis* have been previously described [13]. Here, we confirmed that ulcers in dogs are very similar to the ulcers found in human CL cases with *L. braziliensis,* which supports previous data showing epidemiologic and demographic differences between SC infection and CTL. While the prevalence of SC infection in dogs was similar in males and females, the prevalence of CTL was higher in males than in females. In humans, ATL is more common in males, which is explained by the greater exposure of men to the infection, as their work is more likely to involve agriculture and forest activities [27,31,32,33]. However, as the dogs of the present study were domestic, other factors may be related to the greater susceptibility of males than females in developing leishmaniasis. The same pattern, a greater susceptibility of males than females to develop leishmaniasis, was also documented in children with VL caused by *L. infantum* [34,35]. Of note, VL was also more present in BALB/C and C57BL/6 males than females. While C57BL/6 males expressed more IL-10 and TNF, cytokines linked to disease exacerbation, female expressed predominantly IFN-γ, a cytokine associated with protection [36]. Moreover, the parasite load was higher in male than in female BALB/C mice upon infection with *L. infantum* [36]. The predominance of ulcers mainly on the ears and the scrotum is interesting, as these are cold areas of the body, and in vitro studies indicate that *Leishmania* sp. survive and proliferate in temperatures below 35 °C [37,38].

The long duration of the disease in dogs differs from what has been observed in humans with *L. braziliensis*. Based on clinical trials in which no therapy or placebos were administered to patients with CL, skin ulcers caused by *L. braziliensis* usually healed within one year [13,39,40]. However, in 57.6% of dogs the ulcers remained active for more than a year, and some animals had the disease for more than five years, suggesting that dogs maintain ulcers for a longer period of time. This indicates that self-healing CL in dogs is less frequent than in humans, with 22.7% of dogs followed up in this study. It is important because the presence of dogs with an active disease may influence the number of infected vectors and increase the probability of transmission of the infection.

The high prevalence of canine and human ATL in the village of Nova Esperança, an area where the first cases of CL were detected in 2015, supports the notion that outbreaks of human ATL and CTL are occurring in new areas in the southeast region of the state of Bahia, Brazil. While only a few wild animals have been documented with *L. braziliensis* infection, as well a few cats and horses, based on our observations here and previous studies, dogs may be considered the most important reservoir of *L. braziliensis* [13,41,42,43,44,45]. In these endemic areas of the southeast region of Bahia, dogs are not only prized animals but have an economic importance since they are traded, and their prices vary depending on the ability of dogs to participate in hunting activities of wild animals that are consumed as food. A strong indication that dogs are a primary vector in the *L. braziliensis* transmission chain was documented here. The frequency of people who developed ATL living in homes that had dogs with CTL or animals with SC infection was fourfold higher than in homes without dogs or with dogs infected with *L. braziliensis*.

We recognize that this study has several limitations. The loss in the follow-up of dogs with CTL was about 20%, although this is less than that which has been reported in other studies [30,46]. In a large percentage of dogs, the diagnosis of CTL was made based on the clinical findings plus the detection of antibodies against SLA as the PCR was negative in roughly 30% of the animals with CTL. However, we have shown that CL ulcers in dogs are very similar to the ulcers found in humans with CL, and detection of antibodies to SLA by ELISA is highly sensitive. The percentage of dogs with CTL with positive PCR was similar to the percentage observed in our previous studies [13]. It cannot be ruled out that the low sensitivity of the PCR in CTL may because 99% of the DNA obtained from the biopsies are from the host and not from Leishmania. Additionally, we have shown in humans that the duration of the disease is inversely correlated with the presence of parasites in the lesion [47]. As dogs remain with the disease for years, it is likely that the low number of parasites remaining at the lesion site results in a higher chance of a negative PCR. We also recognize that an important addition to this work would be the documentation that the vector could acquire the infection from sick or infected dogs. We maintained contact with possible collaborators who are entomologists, but none of them had a colony of *N. intermedia* or *N. whitmany* available, and we failed in our attempt to maintain even for a short period of time colonies of phlebotomies captured in this endemic area to perform xenodiagnoses.

There is no recommendation from the Brazilian government about the management of CTL, but we and others have shown that dogs respond very well to intralesional Glucantime [48,49,50]. As this study showed, dogs are a domestic reservoir of *L. braziliensis*, and epidemiologic evidence has pointed out that dogs may participate in the transmission cycle of *L. braziliensis.* From this point of view, the treatment of CTL should be considered as it may not only decrease the suffering of dogs with the disease and reduce the number of these animals that are sacrificed but may also decrease the infection transmission rate and the occurrence of tegumentary leishmaniasis in humans and dogs.

## 5. Conclusions

Despite these limitations, our epidemiologic, clinical, and diagnostic tools clearly indicate that the dog is an important reservoir of *L. braziliensis* in the development of CTL. They remain with the ulcers for a long period of time, and there is an association between dogs with CTL and SC *L. braziliensis* and the occurrence of human CL.

## Figures and Tables

**Table 1:** Demographic and clinical characteristics of dogs with canine tegumentary leishmaniasis and subclinical infection.

	Canine Tegumentary Leishmaniasis	Canine Subclinical Infection	*p* Value
	N = 66	N = 76	
Age (years), mean, (SD)	5 ± 3	4 ± 3	0.09
% of Male	52/66 (79%)	42/76 (55%)	0.03
Duration of illness (days), IQ	90 (30–210)	-	
Positive Serology	66 (100%)	76 (100%)	-
Positive PCR	47 (71%)	3 (3.9%)	0.01
Revaluation after 1 year			
Prevalence of SC *L. braziliensis* Infection	-	76 (35%)	
Active CL	38 (57.6%)	0 (0%)	
Dead or sacrificed dogs	13 (19.7%)	-	
Self-healing CTL	15 (22.7%)	-	

**Table 2:** Canine and human population in the village of Nova Esperança.

	(*n*)
Population of the Village	504
Prevalence of human cutaneous leishmaniasis	183 (36%)
Number of Dogs in the Village	88
Prevalence of dogs with SC *L. braziliensis* infection	20 (22.7%)
Prevalence of canine tegumentary leishmaniasis	13 (14.7%)

## Data Availability

No new data were created or analyzed in this study. Data sharing is not applicable to this article.
